# Exploring the relationship between perceived social support and pregnant women’s physical activity behavior: a chain-mediated effect test

**DOI:** 10.1186/s12884-025-08482-3

**Published:** 2025-12-12

**Authors:** Min Li, Fang Rong Wang, Liang Hui Xu, Yan Jiao Wang, Yan Li

**Affiliations:** 1 School of Nursing, Kunming Medical University, No. 1168, Chunrong West Road, Chenggong District, Kunming City, Yunnan Province China; 2 The Department of obstetrics, The First Affiliated Hospital of Kunming Medical University, No. 295 Xichang Road, Wuhua District, Kunming City, Yunnan Province China; 3 The Department of psychiatry, The First Affiliated Hospital of Kunming Medical University, No. 295 Xichang Road, Wuhua District, Kunming City, Yunnan Province China

**Keywords:** Pregnant women, Perceived social support, Physical activity health belief, Exercise self-efficacy, Physical activity

## Abstract

**Objective:**

This study aims to explore the impact of perceived social support on pregnant women’s physical activity behaviors, as well as the mediating effects of physical activity health beliefs and exercise self-efficacy.

**Methods:**

We selected 283 pregnant women from a hospital in Kunming City from March 2025 to June 2025 using the convenient sampling method. Various scales and questionnaires assess pregnant women’s social support for physical activity, physical activity, health beliefs in physical activity, and exercise self-efficacy. Statistical analysis was performed using SPSS and the bootstrap method to test the chain mediating effect.

**Results:**

(1)Perceived social support can predict physical activity, health beliefs in physical activity, and exercise self-efficacy. Health beliefs in physical activity can predict exercise self-efficacy. Similarly, perceived social support, health beliefs in physical activity, and exercise self-efficacy can positively predict autonomous fitness behavior. (2)The indirect effect of the path with health beliefs in physical activity as the mediating variable is 0.411. The indirect effect of the path with exercise self-efficacy as the mediating variable is 0.352. The indirect effect of the path with health beliefs in physical activity and exercise self-efficacy as mediating variables is 0.383. (3)The sum of all indirect effects is 1.146, and the effects of the three indirect pathways account for 35.8%, 30.7%, and 33.4% of the total respectively.

**Conclusion:**

Perceived social support predicts pregnant women’s physical activity behavior through both the independent mediating effects of physical activity health beliefs and self-efficacy, as well as their sequential chain mediation effect. The research delineates the association patterns and mechanistic pathways, advancing the evidence base regarding the impact of perceived social support on gestational physical activity behaviors. It provides actionable insights for healthcare administrators to optimize prenatal physical activity support systems.

## Introduction

According to global health statistics, prolonged sitting and physical inactivity have become the fourth-leading risk factor for mortality worldwide [1]. To address this public health challenge, China has issued Healthy China 2030 Plan [2], which advocates strengthening the “integration of sports and medicine” model for disease management and health services, and promoting physical activities among key populations. As a special group in society, pregnant women have seen their physical activity levels gradually become a key focus of healthcare providers in China.

Physical activity during pregnancy refers to any movement involving skeletal muscles that increases energy expenditure beyond the resting level [3]. The World Health Organization (WHO) recommends that [4] pregnant women without contraindications to exercise should achieve a standard of 150 min of moderate-intensity physical activity per week. Scientific and appropriate physical activity during pregnancy can yield substantial health benefits in both physical and psychological aspects [5], and extend these benefits to the time of childbirth and the postpartum period [6]. Despite the clear benefits, the level in most regions remains relatively low. Data have been reported from regions including Africa, Europe, Oceania, the Americas, and so on [7]. In China, only 21% of pregnant women can maintain sufficient physical activity [8]. Existing studies have shown that pregnant women are aware of the benefits of physical activity during pregnancy, yet they still fail to engage in such activity [9]. This gap between knowledge and practice makes research on its underlying mechanisms necessary.

To explore the underlying mechanisms, it is necessary to consider the social and cultural contexts that influence pregnant women’s decision-making. Most Chinese people prefer communal living that includes their parents, especially during special periods like pregnancy. This type of family structure makes pregnant women’s decision-making regarding physical activity vulnerable to the influence of their spouses, parents, and other family members [10]. Among these family members, elders such as mothers or mothers-in-law may hold traditional beliefs that prioritize experience over science. They are the main source of misunderstandings that physical activity during pregnancy leads to miscarriage, premature birth, and other adverse outcomes [11], and may also regard bed rest as a way to protect both the mother and the fetus. These perceptions influence pregnant women’s physical activity behavior, and some pregnant women choose to stop or reduce their physical activity once they become pregnant [12]. Additionally, while some other pregnant women can recognize this as a misconception, they only engage in low-level physical activity such as walking due to the invisible pressure from their family environment [13]. Against this family background, positive and scientific social support from external sources is particularly important.

As a key solution, social support comes from sources including medical professionals, family members, community organizations, and other pregnant women. Medical professionals can help pregnant women and their family members establish correct health beliefs by providing scientific guidance on physical activity during pregnancy and health education [14]. When family members, friends, and others hold a positive attitude toward physical activity during pregnancy, this positive feedback can enhance pregnant women’s exercise self-efficacy [15], which is conducive to the formation of sustainable and healthy exercise habits. Therefore, exploring the relationship between perceived social support and pregnant women’s physical activity behavior is an important way to improve their participation in physical activity. Current studies mostly analyze the correlation between individual variables and physical activity separately [16]. There is still a lack of exploration on the internal potential mechanisms and behavioral pathways between the two relationships under a unified theoretical framework, making it difficult to reveal how various factors affect physical activity behavior directly or indirectly.

To deeply explore how social support influences physical activity behavior, this study introduces Self-Determination Theory as the research framework. Self-Determination Theory is a crucial framework for studying the internal mechanisms of health behaviors [17]. It identifies three core needs that determine human behavior, namely the need for relatedness (a sense of belonging and connection), the need for competence (a sense of mastery and ability), and the need for autonomy (a sense of efficacy and ownership over one’s own behavior).When these needs are met, individuals’ health behaviors will become more autonomous [18]. In this study, support from medical staff, family members, and other sources serves as a foundation and can meet pregnant women’s need for relatedness. Healthcare providers or family members support pregnant women’s need for an overall judgment that they can benefit from and perform well in physical activity by providing information on the risks and benefits of insufficient physical activity and emphasizing their ability to engage in such activity, thereby achieving the goal of meeting pregnant women’s need for competence [19]. Meanwhile, the sense of self-efficacy of pregnant women as autonomous individuals can be enhanced by designing forms of exercise that they prefer [19], thereby meeting their need for autonomy. Health beliefs and exercise self-efficacy, as the specific cognitive manifestations of the need for competence and the need for autonomy, constitute two key variables through which social support influences physical activity. The three factors work together to promote pregnant women to transform external social support into internal self-motivation, thereby facilitating sustained and autonomous physical activity behavior.

Therefore, our study proposes three hypotheses: Hypothesis 1: Perceived social support positively predicted physical activity engagement among pregnant women; Hypothesis 2 is proposed: Perceived social support influences pregnant women’s physical activity behavior by enhancing their health beliefs about physical activity; Hypothesis 3 is proposed: Perceived social support influences pregnant women’s physical activity behavior by enhancing their exercise self-efficacy; Hypothesis 4 is proposed: Health beliefs about physical activity and exercise self-efficacy play a chain mediating role between perceived social support and physical activity behavior. This study aims to explore the relationship between pregnant women’s perceived social support and physical activity behavior, as well as the mediating mechanisms therein, based on Self-Determination Theory.

## Methods

### Participants and Procedure

Pregnant women who attended prenatal examinations at a Grade A tertiary hospital obstetrics outpatient clinic in Kunming between March 2025 and June 2025 were recruited as study participants through convenience sampling. Inclusion criteria: (a) Age ≥ 18 years old; ༈b༉ Fetal Pregnancy in B-ultrasound Report; ༈c༉ Pregnant women without absolute contraindications to exercise (refer to the “2019 Canadian Guidelines for Physical Activity During Pregnancy” [20]); ༈d༉ Informed consent and voluntary participation in this study. Exclusion criteria: ༈a༉Those receiving professional physical activity guidance (Professional physical activity guidance has constituted a type of professional information support, which will strengthen external motivation and obscure the authenticity of data); ༈b༉Those with cognitive impairment or communication barriers; ༈c༉Those with a history of mental illness.The sample size was determined using G*Power software (version 3.1.9.7) and the linear model calculation method [21]. These parameters include a power level of 0.9, an alpha level of 0.05, a medium effect size of 0.15, and 16 predictors. The sample size required to meet the needs of the multiple regression model is 150. Taking into account a potential inefficiency rate of 20%, the required number of participants has been adjusted to 180. Ultimately, 310 pregnant women were deemed eligible for inclusion in the study. This study has been approved by the Ethics Committee of the First Affiliated Hospital of Kunming Medical University (2025L067), and all subjects have given informed consent and voluntarily participated in this study.

## Measures and instruments

### Social support scale for physical activity in pregnant women

This scale was originally developed from Sallis’ Social Support Scale [22]. In the Chinese context, Xiang and colleagues [23] culturally adapted and validated the scale by incorporating specific characteristics of pregnant women’s physical activity patterns. This instrument was designed to comprehensively assess individuals’ perceptions of social support across multiple domains. It comprised four distinct dimensions: (1) emotional support, (2) informational support, (3) instrumental support, and (4) companionship support, encompassing a total of 24 items. Each item was rated on a 5-point Likert scale. Responses were scored from 1 (“strongly disagree”) to 5 (“strongly agree”), yielding a total possible score range of 24 to 120 points. Higher mean scores indicated greater perceived levels of social support from others. The scale demonstrated good internal consistency, with a Cronbach’s alpha coefficient of 0.95.

### Pregnancy physical activity questionnaire (PPAQ)

The original version of this scale was developed by Professor Chasan-Taber in the United States [24]. Chinese researchers Zhang Yan et al. [25] subsequently translated and culturally adapted the scale into Mandarin. Three items were removed and two new items were added based on China’s sociocultural context, resulting in a final 31-item scale. This questionnaire was designed to assess physical activity during pregnancy, encompassing four domains: (1) household and caregiving activities, (2) transportation, (3) exercise/sports, and (4) occupational activities. The questionnaire demonstrated excellent content validity with an index of 0.940. The test-retest reliability coefficients were as follows: 0.944 for overall activity (including low-intensity and above), 0.961 for low-intensity activity, 0.877 for moderate-intensity activity, 1.000 for vigorous-intensity activity, and 0.911 for sedentary behavior, demonstrating good reliability and validity.

Each activity item included six response options ranging from “none at all” to “≥3 hours per day or ≥ 3 hours per week”, based on differential frequency and duration of participation. Each response option was assigned a specific weighting coefficient, which was multiplied by the metabolic equivalent (MET) value of the corresponding activity. Based on the calculated energy expenditure, the 31 activities were categorized into: (1) sedentary (< 1.5 METs), (2) light-intensity (1.5–2.9 METs), (3) moderate-intensity (3.0–6.0 METs), and (4) vigorous-intensity (> 6.0 METs) activities.

### Health belief scale for physical activity during pregnancy

Yu et al. [26] developed the Exercise Health Belief Scale based on the HBM theory in 2006. Later, Chen et al. [27] revised and developed the Health Belief Scale for Physical Activity in Pregnant Women on this basis, combining it with the 2019 Canadian Guidelines for Physical Activity During Pregnancy. The scale consists of 5 dimensions and 38 items. Among them, there are 6 items for perceived susceptibility, 6 items for perceived severity, 10 items for perceived benefits, 10 items for perceived barriers, and 6 items for behavioral cues. All items were rated on a 5-point Likert scale, yielding a total possible score range of 38 to 190 points. Higher scores indicated stronger health beliefs regarding physical activity among pregnant women. The Cronbach’s α coefficients for each dimension are 0.844, 0.785, 0.975, 0.903, and 0.922, respectively.

### The pregnancy exercise self-efficacy scale (P-ESES)

The original scale was developed by researchers including Kroll et al. [28]. Subsequently, Bland et al. [29] revised the scale according to the characteristics of pregnant women. The revised scale consists of 1 dimension with 10 items. This scale was cross-culturally adapted into Chinese by Yang et al. [30] in 2017, comprising three dimensions: (1) overcoming exercise barriers, (2) overcoming emotional barriers, and (3) overcoming support barriers, with a total of 10 items. Each item was rated on a 5-point Likert scale with response options: “strongly agree,” “agree,” “neutral,” “disagree,” and “strongly disagree. The total score ranges from 10 to 50 points, with 10–20 points indicating a low level, 21–40 points indicating a moderate level, and 41–50 points indicating a high level. The scale demonstrated good reliability and validity, with the overall Cronbach’s alpha coefficient reaching 0.804.

### Research procedures and statistical analysis

1. Data analysis was performed using IBM SPSS 26.0, including descriptive statistics and correlation analyses for social support, physical activity behavior, physical activity health beliefs, exercise self-efficacy, and other variables.
2. To examine potential common method bias, Harman’s single-factor test was employed following the statistical procedure: Analyze → Dimension Reduction → Factor Analysis. All items were included in an unrotated exploratory factor analysis, yielding 12 factors with eigenvalues >1.0. The largest factor accounted for 12.265% of the variance, which was below the 40% threshold recommended by Hair et al. [31] Therefore, no significant common method bias was detected in this study.
3. Multicollinearity diagnosis: If the tolerance is less than 0.1 or the Variance Inflation Factor (VIF) is greater than 10, the independent variables may have multicollinearity. In this study, the tolerance values and VIF values of each variable were 0.455 to 0.912 and 1.096 to 2.200, respectively. This indicates that there is no multicollinearity among the variables.
4. Mediation analyses were conducted using Model 6 of the SPSS PROCESS macro (Hayes, 2013), examining: (a) the direct effect between perceived social support and pregnant women’s physical activity behavior; (b) the mediating roles of physical activity health beliefs and exercise self-efficacy; and (c) the chain mediating effect between perceived social support and physical activity behaviors of pregnant women.

## Results

A total of 310 pregnant women completed the questionnaire survey. After excluding invalid questionnaires (such as regularized responses, missing data, etc.), 283 valid responses were finally included, with a final effective response rate of 91.2%.

### Demographic information

Among them, the average age of the pregnant women was (30.75 ± 3.83) years old. Most pregnant women (62.5%) have a bachelor’s degree. Occupations are mostly clerical workers (40.5%). Household per capita income was evenly distributed, with the majority (30.4%) reporting an average monthly income between 5,000 and 10,000 RMB. 61.1% of the pregnant women reported occasional physical activity habits prior to pregnancy (defined as < 3 sessions per week). The majority of pregnant women participating in this study were primiparous (73.1%). Table 1 describes detailed information in this regard.

**Table 1:** Demographic statistics of pregnant women (*n* = 283)

Characteristic	Frequency(*n*)	Percentage(%)
Age(M ± SD)		
<25	8	2.9%
25-	101	35.7%
30-	140	49.3%
>35	34	12.1%
Education		
Primary school and below	0	0
Junior high school	4	1.4%
Senior high school or technical secondary school	15	5.3%
Junior college	52	18.4%
Bachelor degree	177	62.5%
Master degree or above	35	12.4%
Occupation		
Unemployed	33	11.7%
worker	36	12.7%
Sedentary office workers	115	40.5%
medical staff	28	9.9%
self-employment	30	10.6%
service industry	7	2.5%
teacher	33	11.7%
Other	1	0.4%
Average monthly income(CNY)		
<2000	2	0.7%
2000–3000	6	2.1%
3000–5000	37	13.1%
5000–10,000	86	30.4%
10,000–15,000	78	27.6%
>15,000	74	26.1%
Whether there was a habit of doing physical exercise before pregnancy		
None	59	20.8%
Occasionally, less than 3 times a week	173	61.1%
Frequently, 3–5 times a week	51	18%
Obstetric history		
Primipara	207	73.1%
Multipara	76	26.9%

### Statistical description and correlation analysis

First, this study used the Shapiro-Wilk test to conduct a normality test on all continuous variables. The results showed that the P-value of each variable was < 0.05. Therefore, the median (M) and interquartile range (IQR) were used to describe the data distributions of social support, physical activity behavior, physical activity health beliefs, and exercise self-efficacy, which indicates that none of the variables conformed to a normal distribution. Based on this, this study adopted Spearman’s rank correlation coefficient to analyze the strength of the association between variables such as physical activity during pregnancy, social support, and family values. The significance test level for the correlation coefficient was set at α = 0.05. (See Table 2 for results)

Because the data is skewed distributed, Table 2 shows the median values, interquartile ranges, and correlation coefficients for perceived social support, physical activity behavior, physical activity health beliefs, and exercise self-efficacy. The results indicate that the correlations between variables have reached statistically significant levels. Among these, perceived social support showed statistically significant positive correlations with physical activity, physical activity health beliefs, and exercise self-efficacy (*p* < 0.01).

**Table 2:** Descriptive statistical results for each variable and the correlation matrix (r) of variables

Variables	Median, quartile	1	2	3	4
1Perceived social support	(90, 106)	1			
2Physical activity	(129.85, 258.07)	0.351**	1		
3Health beliefs about physical activity	(135, 169.35)	0.270**	0.736**	1	
4Exercise self-efficacy	(34, 41.35)	0.341**	0.783**	0.653**	1

*P < 0.05, **P < 0.001, the same below

### Significance testing of mediation effects

In this study, Model 6 of the SPSS PROCESS macro developed by Hayes (2013) was employed to examine the mediation effects. Variables such as age, occupation, and obstetric history were not included in the final model. Because they had no significant association with the dependent variable (*P* > 0.1), and pre-analysis showed that their impact on the core path coefficients was less than 5%; excluding them helped avoid excessive complexity of the model. As shown in Table 3, perceived social support directly predicted pregnant women’s physical activity behavior (β = 0.650, *p* = 0.005), supporting Hypothesis 1. Perceived social support positively predicted physical activity health beliefs༈β = 0.586, *p* < 0.001༉. Perceived social support also positively predicted exercise self-efficacy༈β = 0.077, *p* = 0.001༉. Physical activity health beliefs directly and positively predicted exercise self-efficacy༈β = 0.143, *p* < 0.001༉. Perceived social support, physical activity health beliefs, and exercise self-efficacy collectively and positively predicted physical activity behavior༈β = 0.650, *p* = 0.005; β = 0.702, *p* < 0.001; β = 4.567, *p* < 0.001༉.

**Table 3:** Regression analysis of the relationship between variables

Effect	Item	Effect	SE	t	*P*	95% confidence interval
Direct effect	Perceived social support → Physical activity	0.650	0.230	2.827	0.005	[0.197, 1.103]
Indirect effect	Perceived social support → Health beliefs about physical activity(M1)	0.586	0.156	3.753	< 0.001	[0.278, 0.893]
	Perceived social support → Exercise self-efficacy(M2)	0.077	0.023	3.347	0.001	[0.032, 0.122]
	Health beliefs about physical activity(M1)→ Exercise self-efficacy༈M2༉	0.143	0.009	16.417	< 0.001	[0.126, 0.161]
	Health beliefs about physical activity(M1)→ Physical activity༈Y༉	0.702	0.121	5.804	< 0.001	[0.464, 0.940]
	Exercise self-efficacy(M2)→ Physical activity	4.567	0.596	7.663	< 0.001	[3.393, 5.740]
Total effect	Perceived social support → Physical activity	0.664	0.229	2.894	0.004	[0.212, 1.116]

**Fig. 1:**
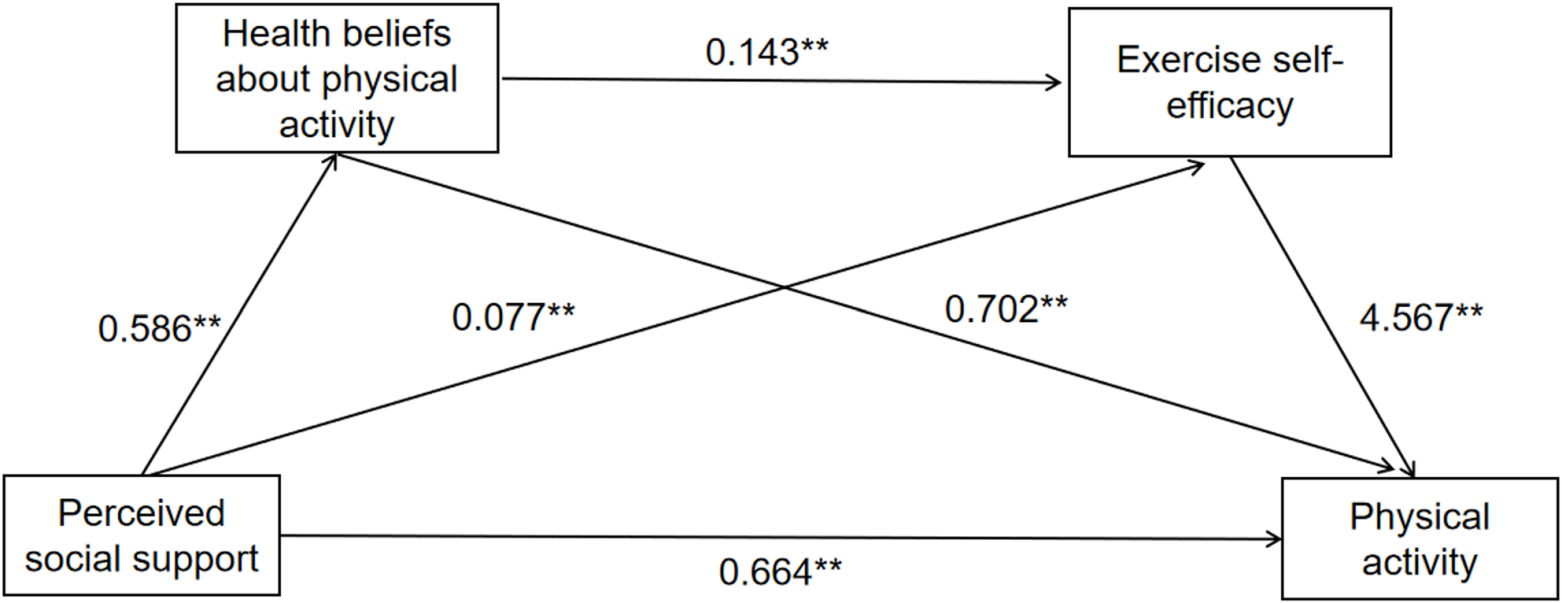
The chain-mediated pathway of perceived social support on maternal physical activity behavior was significant. **p < 0.01, *p* < 0.001

Results of further serial mediation model tests are presented in Table 4 and illustrated in Fig. 1. The indirect effect through the pathway with physical activity health beliefs as the mediator was 0.411(95% CI = [0.142, 0.761]). The indirect effect through the pathway with exercise self-efficacy as the mediator was 0.352༈95% CI = [0.132, 0.606]༉. The indirect effect through the serial mediation pathway with physical activity health beliefs and exercise self-efficacy as mediators was 0.383༈95% CI = [0.146, 0.687]༉. The total sum of all indirect effects was 1.146༈95% CI = [0.599, 1.785]༉. The three indirect pathways accounted for 35.8%, 30.7%, and 33.4% of the total effect, respectively. Therefore, a serial mediation effect of perceived social support on pregnant women’s physical activity behavior was established.

**Table 4:** Bootstrap analysis of significance test of intermediary effect

Influence path	Indirect effect		95% confidence interval		
		BootSE	BootLLCI	BootULCI	The ratio of total effect
TOTAL	1.146	0.299	0.599	1.785	
Ind1	0.411	0.159	0.142	0.761	35.8%
Ind2	0.352	0.121	0.132	0.606	30.7%
Ind3	0.383	0.135	0.146	0.687	33.4%
(C1)	0.059	0.205	−0.325	0.488	
(C2)	0.027	0.130	−0.226	0.305	
(C3)	−0.032	0.161	−0.363	0.265	

Ind1:Perceived social support → Health beliefs about physical → Physical activity

Ind2:Perceived social support → Exercise self-efficacy → Physical activity

Ind3:Perceived social support → Health beliefs about physical → Exercise self-efficacy → Physical activity

## Discussion

Our study demonstrates significant positive correlations among perceived social support, physical activity-related health beliefs, exercise self-efficacy, and physical activity behavior. The effects of these variables on physical activity participation among pregnant women have been empirically validated. The data analysis results confirm the validity of all hypotheses proposed in this study.

This study shows that the physical activity energy expenditure of pregnant women is 140.63 ± 62.49 Met·h/week. According to the World Health Organization (WHO) standards for activity intensity, the compliance rate reaches 54.41%. It is higher than the results of some previous studies conducted abroad [32] and in China [33, 34]. The possible reasons for this analysis are as follows: at the social level, the vigorous promotion of a number of policies in China in recent years has provided a favorable social environment for physical activity during pregnancy, and promoted the transformation of awareness of physical activity during pregnancy to practice from the perspective of the external environment [10]. At the individual level, the participants in this study are mostly primiparas with a good education background, who have a better understanding of scientific concepts and stronger willingness and ability to practice healthy behaviors [35]. This finding also suggests that future attention should be paid to the cultural and cognitive barriers that pregnant women with low educational levels may face. Perceived social support can positively predict the physical activity behavior of pregnant women, which verifies Hypothesis 1. This is consistent with the research of other scholars [36, 37]. Healthcare providers should maximize the activation of pregnant women’s social support systems. Shum [16] indicated that encouragement from family members, especially spouses, can enhance pregnant women’s enthusiasm for physical activity. Therefore, while providing support to pregnant women, healthcare providers should also focus on activating family members’ motivation to participate. Cheng [38] found that online support interventions have potential benefits for the management of gestational diabetes mellitus (GDM), and most pregnant women hold a supportive attitude towards them. Healthcare providers can leverage mobile health to enhance the accessibility of prenatal care knowledge [39]. In the future, novel support models such as gamified digital interventions [40] and virtual technologies [41] can also be actively explored to promote pregnant women’s active participation in physical activity.

### The mediating role of health beliefs in physical activity

This study shows that the health belief regarding physical activity plays a mediating role in the relationship between perceived social support and pregnant women’s physical activity behaviors, which verifies Hypothesis 2 of this study. This is consistent with the findings of Tyler’s study [42]. The study by Wang et al. [37] focused on the mediating effect of fear of falling in pregnant women with GDM, and also confirmed that social support needs to indirectly influence physical activity behavior through cognitive-level variables. This conclusion can be explained by the Health Belief Model. This theory proposes that individuals’ health cognition plays a key bridging role between the social environment and health behaviors [43]. As Glavaš D [44] indicated, the belief that physical activity is beneficial can yield significant benefits in terms of health outcomes. Based on this, medical staff can break down the cognitive barriers formed by traditional empiricist notions through professional support, correctly view the unreasonable parts in traditional concepts, and enhance the subjective recognition of behavioral benefits [45]. However, the current lack of advice from healthcare professionals may be a barrier factor [46]. This can be improved by optimizing the allocation of human resources and enhancing one’s own professional capabilities. Healthcare providers can proactively design and implement intervention measures based on the Health Belief Model. Previous studies have confirmed that [47] interventions within this framework can effectively enhance health beliefs and provide a practical pathway for pregnant women to improve their physical activity levels.

### The mediating role of exercise self-efficacy

This research findings indicate that exercise self-efficacy plays a mediating role in the relationship between perceived social support and physical activity behaviors among pregnant women. This finding also provides validation for Hypothesis 3. This is consistent with the research findings of Zhao et al. [45]. Chen et al. [48], in their study on pregnant women with gestational diabetes mellitus, also confirmed this result. As a strong predictor of physical activity, self-efficacy plays an important role in predicting and maintaining physical activity behavior [49]. Pregnant women are prone to doubting their own activity abilities due to physical and mental changes. Those with higher self-efficacy can better regulate themselves [50], transform activity intentions into practice and persist in it. It is worth noting that the maintenance of self-efficacy is characterized by dynamic regulation [51]. Pregnant women face different challenges during different stages of pregnancy, which may weaken their self-efficacy in phases. At this point, sustained social support can consolidate confidence and prevent behavioral disruption caused by a decline in self-efficacy. This suggests that healthcare workers should pay attention to the importance of self-efficacy as a mediating variable and select intervention measures that are easily acceptable to pregnant women and meet their cultural needs to improve self-efficacy. Previous studies have combined family supervision, structured peer education, and mobile health [52] for pregnant women with gestational diabetes mellitus (GDM), which has improved their exercise self-efficacy. In the future, such comprehensive intervention models can be promoted to a broader group of general pregnant women.

### The chain mediating role of health belief in physical activity and exercise self-efficacy

This study further found that perceived social support can indirectly predict pregnant women’s physical activity behaviors through the chain mediating effect of physical and mental activity health beliefs and exercise self-efficacy. This verifies Hypothesis 4. This has not been mentioned in previous articles. External support changes pregnant women’s cognitive framework regarding physical activity through information transmission and value enhancement [53]. This support-driven transformation of beliefs enables pregnant women to develop non-traditional rational judgments, laying a cognitive foundation for the subsequent establishment of self-efficacy. This is the role of belief as the first link in the mediation process. In this process, healthcare providers play an important role [54]. Studies have shown [55] that the intervention method using barrier belief counseling has achieved effective improvement in physical activity. We should correctly assess the health beliefs of pregnant women and their family members, transform their perceptions through professional counseling, analyze barrier factors, and shape scientific health beliefs for pregnant women.

Based on health beliefs, exercise self-efficacy further transforms cognition into conviction in one’s own abilities. This transformation constitutes the second link in the mediating effect. Social support can not only act directly on behavior, but also pave the way for the initiation and maintenance of behavior by changing the cognition of traditional thinking and enhancing self-efficacy. This pathway is also consistent with the idea of Self-Determination Theory that converts external support into internal self-motivation [56]. Healthcare providers should attach importance to the social support network of pregnant women and take into account the dual targets of beliefs and self-efficacy during intervention. It is necessary not only to strengthen health beliefs through multi-dimensional support, but also to enhance self-efficacy through step-by-step successful experiences, ultimately promoting pregnant women to actively engage in physical activity behaviors.

### Implications for future nursing practice

Healthcare providers should implement the national concepts and actively promote the implementation of policies. In future practice, it is necessary to coordinate society, hospitals, communities, and families to create a positive social environment for pregnant women. Obstetric staff should fully mobilize social support networks including family members and peers, incorporate families into health belief assessment and intervention, adopt family-oriented health education to correct cognitive misunderstandings and popularize knowledge about physical activity, and guide family members to participate in and provide support. Secondly, personalized physical activity consultation and continuous follow-up should be provided to help pregnant women develop strategies to cope with changes during pregnancy and enhance their self-efficacy. When designing intervention measures, the cultural background of pregnant women should be assessed, and traditional concepts should be integrated with modern health concepts to ensure the acceptance of the measures by pregnant women and their cultural adaptability. Finally, regular assessment and feedback mechanisms should be adopted to help pregnant women shift from passively receiving support to proactively maintaining healthy physical activity behaviors, thereby promoting the health of both mothers and infants.

### Limitations and suggestions for future research

This study has several limitations. This study adopted a convenience sampling strategy from a single hospital. The sample source was singular and exhibited homogeneity in structure, with participants mainly concentrated in the group of primiparas with good education. This limits the overall extrapolation and generalizability of the research results to a broader population of pregnant women. Furthermore, this study adopted a cross-sectional design, and all data were collected at a single time point. This prevents the establishment of long-term longitudinal dynamic associations between variables. In addition, this study used a self-report method with paper questionnaires for measurement, which is susceptible to factors such as social desirability bias and recall bias, leading to discrepancies between the reported data and actual physical activity behaviors. In the future, efforts should be made to expand the sample sources, achieve adequate coverage of pregnant women from different subgroups as much as possible, and adopt more objective measurement methods such as accelerometers or fitness trackers. This is to verify the accuracy of the data and determine whether the conclusions can be widely applied to other pregnant women. During the research design phase, longitudinal design or interventional design can be adopted, and subgroup analysis can be conducted to explore differences in the relationships between variables across different groups. This approach aims to reveal the dynamic influencing mechanisms of pregnant women’s physical activity behaviors, derive more detailed conclusions, and provide a basis for personalized health management for pregnant women.

## Conclusion

Perceived social support can independently predict the physical activity behaviors of pregnant women. Health beliefs in physical activity and exercise self-efficacy play an important mediating role between perceived social support and physical activity. There are three mediating pathways: the independent mediating role of health beliefs in physical activity, the independent mediating role of exercise self-efficacy, and the chain mediating role of health beliefs in physical activity and exercise self-efficacy. Therefore, families, hospitals, society, etc., should provide the social support that pregnant women need, perceive and accept. Healthcare providers should take corresponding measures to improve pregnant women’s self-cognition. It can not only enhance health beliefs in physical activity but also improve pregnant women’s exercise self-efficacy, thereby promoting their physical activity behaviors.

## Data Availability

The data sets used and/or analyzed during the current study are not publicly available due to confidentiality of data and subsequent research, but are available from the corresponding author on reasonable request.
